# 7-Ketocholesterol induces ATM/ATR, Chk1/Chk2, PI3K/Akt signalings, cytotoxicity and IL-8 production in endothelial cells

**DOI:** 10.18632/oncotarget.12578

**Published:** 2016-10-11

**Authors:** Mei-Chi Chang, Yi-Jane Chen, Eric Jein-Wein Liou, Wan-Yu Tseng, Chiu-Po Chan, Hseuh-Jen Lin, Wan-Chuen Liao, Ya-Ching Chang, Po-Yuan Jeng, Jiiang-Huei Jeng

**Affiliations:** ^1^ Biomedical Science Team, Chang Gung University of Science and Technology, Kwei-Shan, Taoyuan, Taiwan; ^2^ Department of Dentistry, Chang Gung Memorial Hospital, Taipei, Taiwan; ^3^ School of Dentistry and Department of Dentistry, National Taiwan University Medical College and National Taiwan University Hospital; ^4^ Department of Dentistry, Show Chwan Memorial Hospital, Changhua, Taiwan; ^5^ Department of Dentistry, Mackey Memorial Hospital, Taipei, Taiwan

**Keywords:** apoptosis, atherosclerosis, cytotoxicity, endothelial cells, inflammation, Gerotarget

## Abstract

Cardiovascular diseases (atherosclerosis, stroke, myocardiac infarction etc.) are the major systemic diseases of elder peoples in the world. This is possibly due to increased levels of oxidized low-density lipoproteins (oxLDLs) such as 7-ketocholesterol (7-KC) and lysophosphatidylcholine (LPC) that damage vascular endothelial cells, induce inflammatory responses, to elevate the risk of cardiovascular diseases, Alzheimer's disease, and age-related macular degeneration. However the toxic effects of 7-KC on endothelial cells are not known. In this study, 7-KC showed cytotoxicity to endothelial cells at concentrations higher than 10 μg/ml. 7-KC stimulated ATM/Chk2, ATR-Chk1 and p53 signaling pathways in endothelial cells. 7-KC also induced G0/G1 cell cycle arrest and apoptosis with an inhibition of Cyclin dependent kinase 1 (Cdk1) and cyclin B1 expression. Secretion and expression of IL-8 in endothelial cells were stimulated by 7-KC. 7-KC further induced intracellular ROS production as shown by increase in DCF fluorescence and Akt phosphorylation. LY294002 attenuated the 7-KC-induced apoptosis and IL-8 mRNA expression of endothelial cells. These results indicate that oxLDLs such as 7-KC may contribute to the pathogenesis of atherosclerosis, thrombosis and cardiovascular diseases by induction of endothelial damage, apoptosis and inflammatory responses. These events are associated with ROS production, activation of ATM/Chk2, ATR/Chk1, p53 and PI3K/Akt signaling pathways.

## INTRODUCTION

Cardiovascular diseases (atherosclerosis, stroke, myocardiac infarction etc.) are the major systemic diseases of elder peoples in the world. This is possibly due to increased levels of oxidized low-density lipoproteins (oxLDL) that elevate the risk of cardiovascular diseases. Oxidized low-density lipoprotein (OxLDL) contains mainly lysophosphatidylcholine (LPC), lipid ester-bound aldehyses, 7-ketocholesterol (7-KC), 7α-hydroxycholesterol, 7β-hydroxycholesterol, 5α,6α- epoxycholesterol, 5β,6β-epoxycholesterol, 25-hydroxycholesterol, (25R)-26- hydroxycholesterol), and cholesta-3,5-dien-7-one [1]. Various oxLDLs show differential toxic effects to arotic vascular smooth muscle cells [2]. 7-KC was also a proinflammatory oxysterol present in atherosclerotic plaque and even had more atherosclerotic activity than cholesterol [3]. 7-KC is shown to stimulate various degenerated diseases such as Alzheimer's disease, and age-related macular degeneration [4]. It has been shown to induce oxidative stress and mitochondrial DNA damage to epithelial cells [5] and contribute to the initiation and progression of atherosclerosis [3]. oxLDL also stimulates tissue factor (TF) production of mononuclear cells and macrophages, leading to thrombus formation and atherosclerosis [6]. All these results indicate the crucial role of 7-KC toxicity on human health.

Endothelial cells are important for vascular homeostasis, angiogenesis, wound healing etc. Impairment of endothelial functions is may elevate the risk of a number of diseases including atherosclerosis, thrombosis, tumor metastasis, and diabetes. Inflammatory cell infiltration of vascular walls, reactive oxygen species (ROS) production, generation of oxLDLs, and apoptosis of endothelial cells are involved in the pathogenesis of atherosclerosis [7, 8]. Oxidative stress may activate inflammatory response of endothelial cells and induce the release of various cytokines such as interleukin-1 (IL-1), tumor necrosis factor-α (TNF-α), IL-6, IL-4, chemokines (IL-8 etc.) and cell adhesion molecules (e.g., intercellular adhesion molecule-1 [ICAM-1], E-selectin etc.), leading to infiltration of inflammatory cells through endothelial cells into tissue and contributing to atherosclerosis [9]. OxLDL (< 5 μg/ml) has been shown to stimulate endothelial lectin-like oxidized low-density lipoprotein receptor-1 (LOX-1)/mitogen-activated protein kinases (MAPK)/NF-kB signaling pathway to induce cytokines, metalloproteinases, vascular endothelial growth factor (VEGF), peroxisome proliferator-activated receptor-gamma (PPAR-γ) and LOX-1 expression, thus involve in the progression of angiogenesis/atherosclerosis/carcinogenesis [10]. These oxLDLs are shown to stimulate ROS production, receptor activation, induce signal transduction, cytotoxicity/apoptosis and inflammatory mediators (IL-8, prostanoids, monocyte chemotactic protein-1 [MCP-1] etc) to affect the activities of vascular cells such as endothelial cells, mononuclear cells, and vascular smooth muscle cells, leading to dysfunction and diseases (atherosclerosis, thrombosis, tumor metastasis, diabetes etc.) formation. However the role of 7-KC in stimulating tissue inflammation in vascular walls awaits further investigation.

Level of ROS in tumor tissues and atherosclerotic tissues is higher than healthy tissues [10]. The sources of ROS in endothelial cells are derived mainly from NADPH oxidase, xanthine oxidase, arachidonic acid (AA) metabolism or mitochondrial electron transfer. Antioxidants such as pyrrolidine thiocarbamate (PDTC)□N-acetyl-L-cysteine (NAC), vitamin C, Vitamin E and epigallocatechin gallate (EGCG) inhibit endothelial cells inflammatory response as well as IL-6, MCP-1 and vascular cell adhesion molecule-1 (VCAM-1) expression [9]. NADPH oxidase inhibitor - diphenylene iodonium (DPI) effectively suppress the IL-4-induced ROS production, IL-6 and MCP-1 secretion of endothelial cells [11].

To know the role of 7-KC in the pathogenesis of cardiovascular diseases, the purposes of this study were to investigate the effect of 7-KC on the growth, Ataxia Telangiectasia Mutated Protein (ATM)/Ataxia telangiectasia and Rad3 related (ATR)-cell cycle checkpoint kinase-1 (Chk1)/Chk2, p53, cell cycle kinetics, apoptosis and IL-8 production of endothelial cells. ROS production and protein kinase B (Akt) activation were also studied.

## RESULTS

### Cytotoxicity of 7-KC to endothelial cells

**Figure 1:**
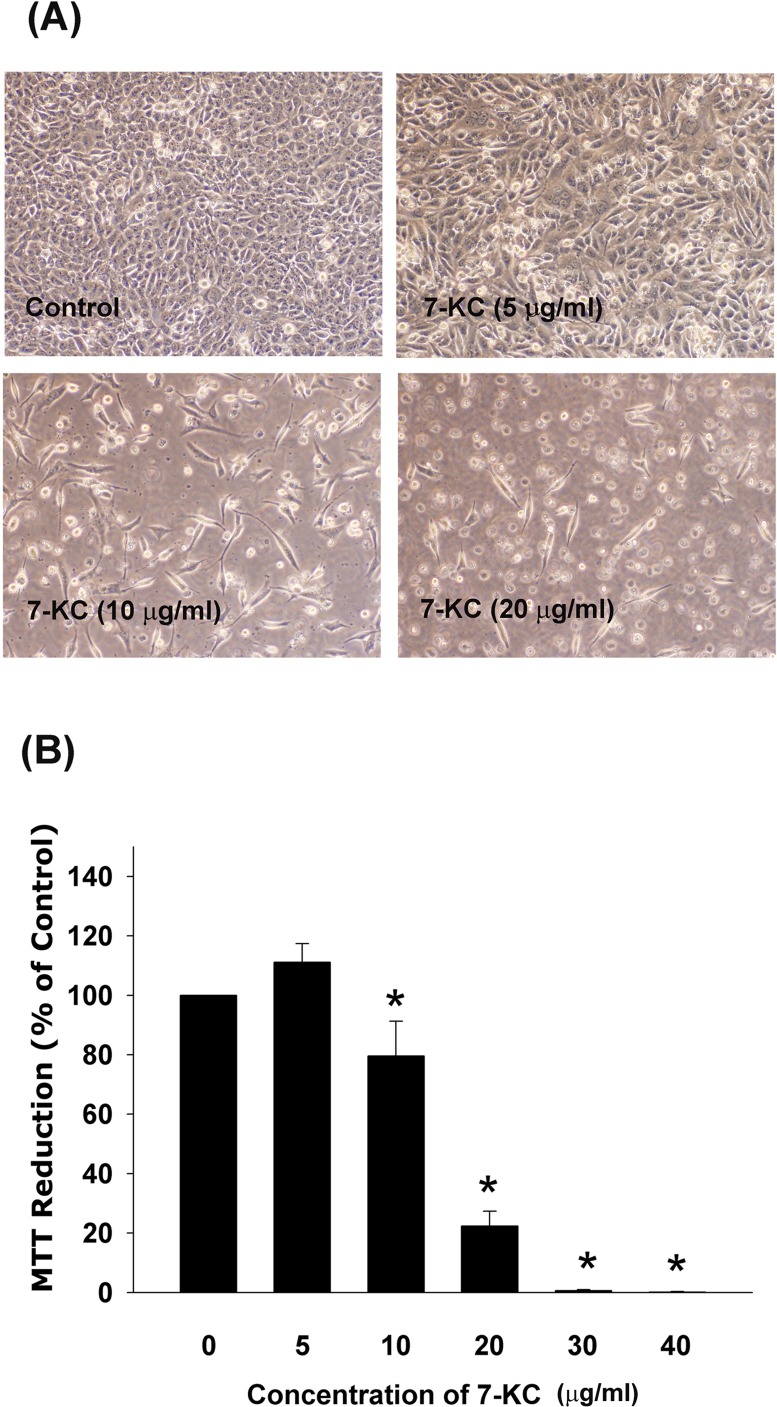
Cytotoxicity of 7-KC (10-40 **μ**g/ml) to endothelial cells after 3 days of exposure **A.** Morphologic changes of EAHY endothelial cells after exposure to different concentrations of 7-KC, **B.** Cyotoxicity of 7-KC to endothelial cells as analyzed by MTT. Results were expressed as Mean ± SE (*n* = 6). *denotes statistically significant difference (*p* < 0.05) when compared with solvent control.

7-ketocholesterol (7-KC) showed cytotoxicity to Eahy926 (EAHY) endothelial cells. Evident morphologic changes of EAHY cells was observed after exposure to 10 and 20 μg/ml of 7-KC (Figure 1A). Quantitatively, cytotoxicity of 7-KC to endothelial cells was noted when the concentations of 7-KC were higher than 10 μg/ml (Figure 1B).

### Induction of cell cycle arrest of endothelial cells by 7-KC

**Figure 2:**
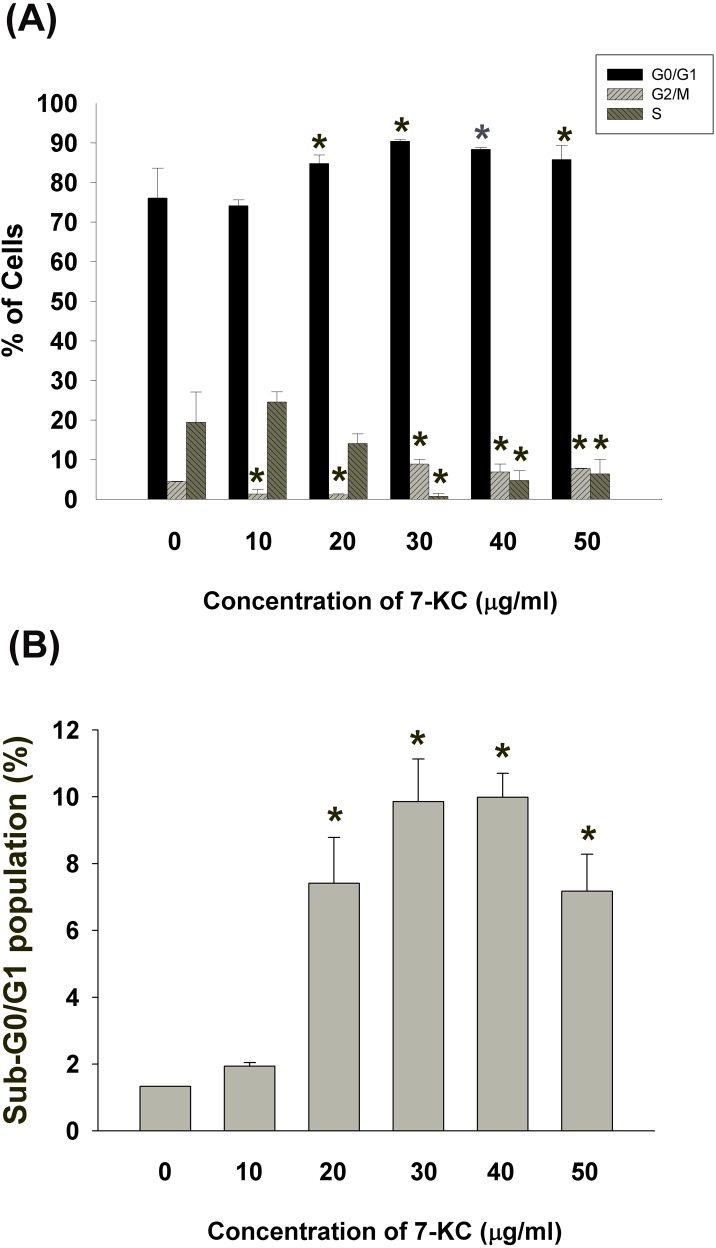
Effect of 7-KC (10-50 μg/ml) on cell cycle progression and apoptosis of endothelial cells **A.** Effect of 7-KC on cell cycle distribution of endothelial cells as analyzed by Modifit Software, **B.** Effect of 7-KC on sub-G0/G1 population of endothelial cells was analyzed by Cell Quest program. Results were expressed as Mean ± SE (*n* = 3).

7-KC also induced cell cycle arrest and apoptosis of EAHY endothelial cells. 7-ketocholesterol (7-KC, > 20 μg/ml) induced G0/G1 cell cycle arrest of endothelial cells. At concentrations higher than 30 μg/ml, 7-KC further induced G2/M cell cycle arrest (Figure 2A). The apoptotic population (sub-G0/G1 population) increased by exposure to different concentrations of 7-KC (Figure 2B).

### Induction the apoptosis of endothelial cells by 7-KC

**Figure 3:**
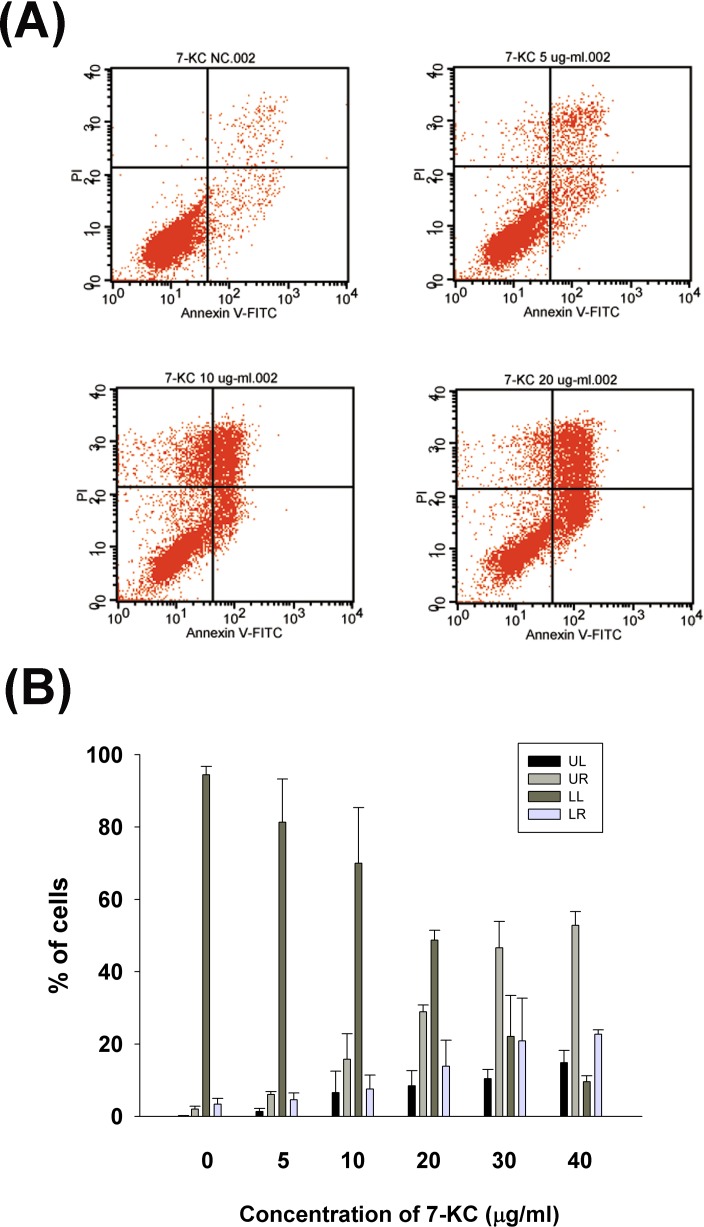
Effect of 7-KC (5-40 μg/ml) on apoptosis of endothelial cells as analyzed by PI and annexin V dual fluorescent flow cytometry **A.** One representative flow cytometry picture was shown. LL (lower left): viable cells, UL (upper left): necrotic cells, LR (lower right): pro-apoptotic cells, UR (upper right): apoptotic cells, **B.** Quantitative analysis of PI + annexin V flow cytometric analysis. Results were expressed as Mean ± SE (*n* = 3).

7-KC induced apoptosis of endothelial cells at concentrations higher than 5 ug/ml as further analyzed and confirmed by propidium iodide (PI)/Annexin V flow cytometric analysis (Figure 3A). Increase in upper right (late apoptosis) and lower right (early apoptosis) population of endothelial cells was observed after exposure to 7-KC at 10 μg/ml or higher (Figure 3A, 3B).

### Effect of 7-KC on cell cycle-related genes and protein expression of endothelial cells

**Figure 4:**
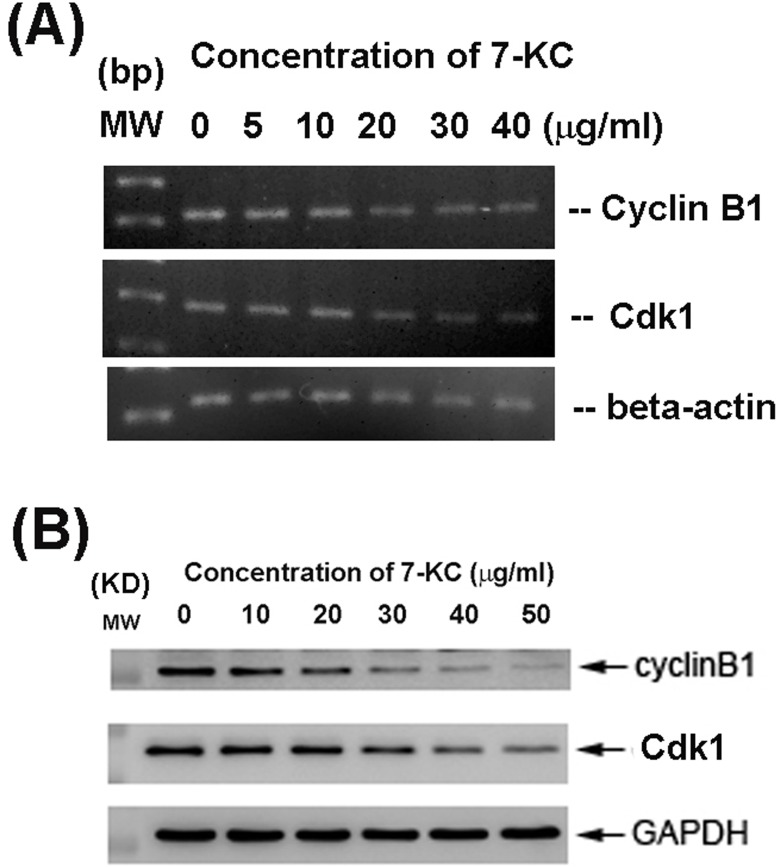
Effect of 24-h exposure to 7-KC on cell cycle-related Cdk1 and cyclin B1 mRNA and protein expression of endothelial cells **A.** mRNA expression of Cdk1 and cyclin B1 as analyzed by PCR. Beta-actin expression was used as control. MW (molecular weight - base pairs [bp]) **B.** Cdk1 and cyclin B1 protein expression as analyzed by western blotting. MW (molecular weight, KD), Expression of beta-actin and GAPDH was used as control for PCR and western blot, respectively. One representative RT-PCR and western blotting result was shown.

7-KC inhibited Cyclin-dependent kinase 1 (Cdk1, also as cdc2) and cyclin B1 mRNA expression of endothelial cells at concentrations higher than 20 μg/ml (Figure 4A). Accordingly, 7-KC also suppressed Cdk1 and cyclin B1 protein expression of endothelial cells at concentrations higher than 20 μg/ml as measured by western blotting (Figure 4B).

### Stimulation the p-ATM, p-ATR, p-Chk1, p-Chk2 and p-p53 Expression of EAHY Cells by 7-KC

**Figure 5:**
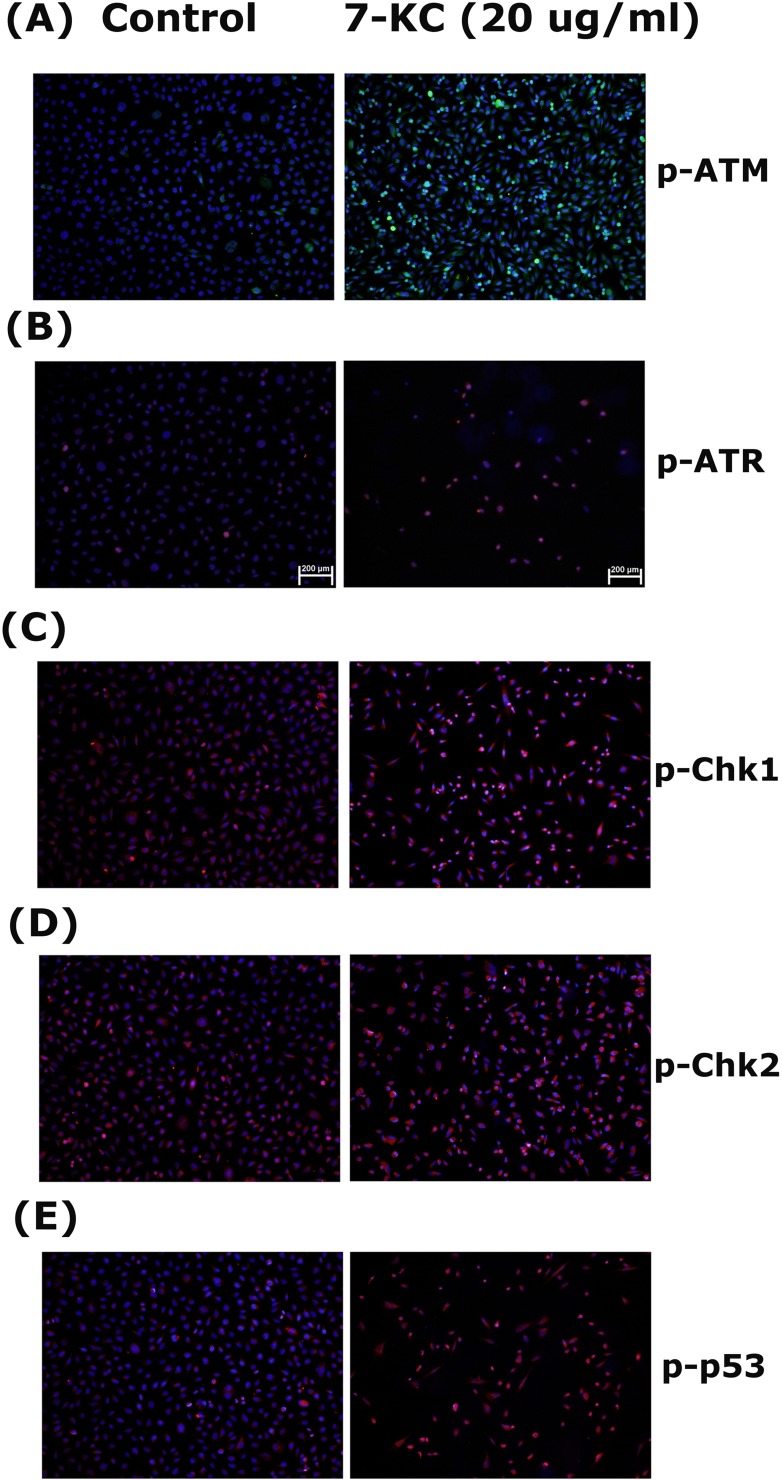
Stimulation of p-ATM, p-ATR, p-Chk1, p-Chk2 and p-p53 expression by 7-KC (20 μg/ml) to endothelial cells EAHY endothelial cells were exposed to solvent control and 20 μg/ml of 7-KC for 24 hours. Immunofluorescent (IF) microscopic observation was done to evaluate the expression of **A.** p-ATM, **B.** p-ATR, **C.** p-Chk1, **D.** p-Chk2 and **E.** p-p53 in endothelial cells. One representative IF picture was shown. (blue - DAPI, red or green - target proteins, p-ATM, p-ATR, p-Chk1, p-Chk2, p-p53)

7-KC (20 μg/ml) stimulated ATM phosphorylation of endothelial cells as revealed by an increase in green fluorescence (Figure 5A, 5B). 7-KC also induced p-ATR, p-Chk2 and p-Chk2 expression of endothelial cells as revealed by an increase in cellular red fluorescence (Figure 5C, 5D). The p53 phosphorylation of endothelial cells was also accelerated after 24 hours exposure to 7-KC (Figure 5E).

### Effect of 7-KC on cytokine secretion of endothelial cells

**Figure 6:**
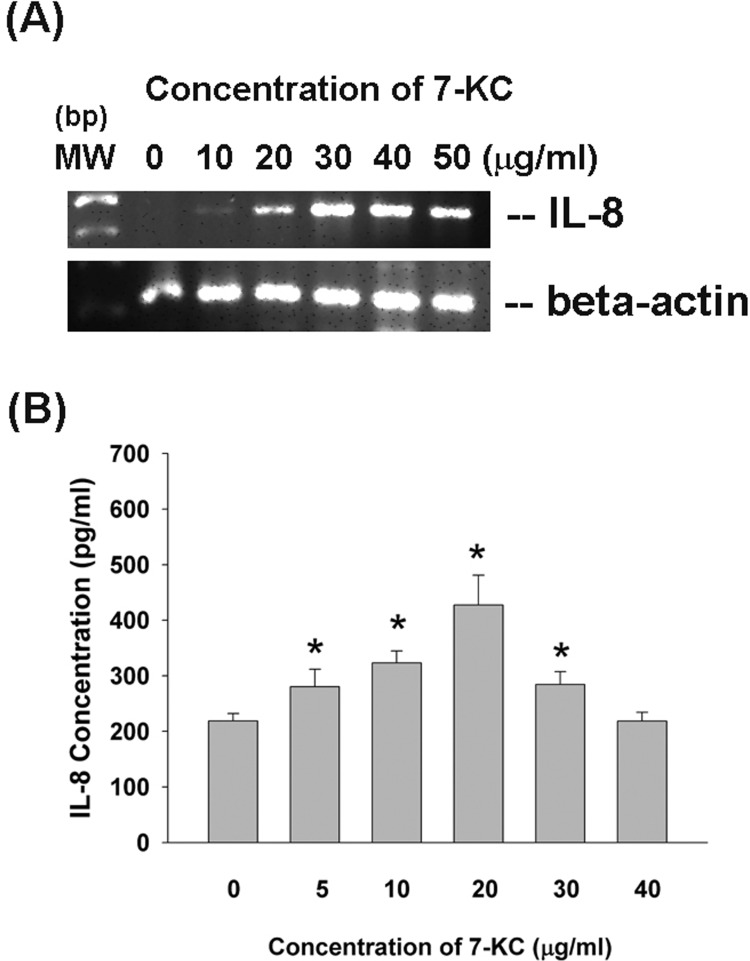
Effect of 7-KC on IL-8 expression and production EAHY endothelial cells were exposed to 7-KC. **A.** Total RNA was isolated and subjected to RT-PCR analysis of IL-8 expression, **B.** Culture medium was collected and used for measurement of IL-8 secretion in endothelial cells. Results were expressed as Mean ± SE (*n* = 5).

7-KC (> 10 μg/ml) stimulated IL-8 mRNA expression of endothelial cells (Figure 6A). In addition, 7-KC (5-30 μg/ml) also induced the secretion of IL-8 in endothelial cells as analyzed by ELISA (Figure 6B), but showed no stimulatory effect on IL-8 secretion by 40 μg/ml of 7-KC possibly due to cytotoxicity.

### Induction of ROS formation and Akt activation of endothelial cells by 7-KC

**Figure 7:**
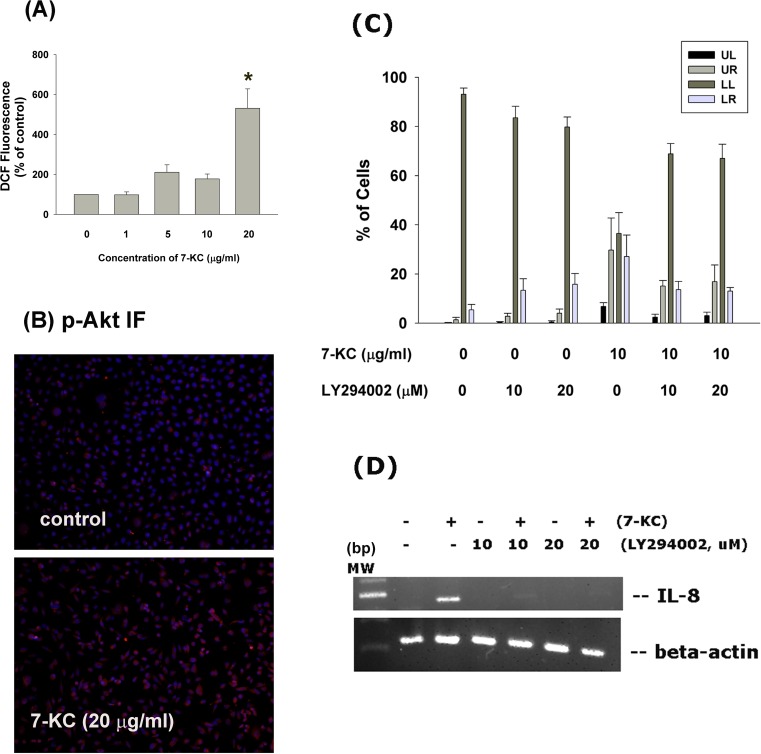
Effect of 7-KC on cellular ROS levels and Akt activation **A.** 7-KC (10-40 μg/ml) induced ROS production as shown by increase in cellular DCF fluorescence. Results were expressed as Mean ± SE (% of control, *n* = 3), **B.** 7-KC stimulate Akt phosphorylation as shown by increase in cellular p-Akt fluorescence (blue - DAPI, red - p-Akt). One representative immunofluorescent study was shown. **C.** LY294002 attenuated the 7-KC-induced apoptosis of endothelial cells as analyzed by PI+Annexin V dual staining flow cytometry. Results were expressed as percentage (% of control, *n* = 6) of cell residing in UL, LL, UR and LR area of flow cytometry chart. **D.** LY294002 prevented the 7-KC-induced IL-8 mRNA expression of endothelial cells.

7-KC (20 μg/ml) induced ROS level of endothelial cells as analyzed by DCF fluorescence flow cytometric analysis. At concentrations of 10 and 20 μg/ml, 7-KC stimulated ROS level to 152% an 185% of control, respectively (Figure 7A). Immunofluorescent staining study showed that 7-KC (20 μg/ml) stimulated Akt phosphorylation of endothelial cells as shown by an increase in p-Akt (red) fluorescence (Figure 7B).

### Role of PI3K/Akt signaling on 7-KC-induced apoptosis and IL-8 expression of endothelial cells

LY294002 (a phosphoinositide 3-kinase [PI3K]/Akt signaling inhibitor) prevented the 7-KC-induced apoptosis of endothelial cells as analyzed by PI and annexin V dual fluorescent flow cytometry (Figure 7C). The percentage of pro-apoptotic cells (LR) and apoptotic (UR) cells decreased after pretreatment and co-incubation of 7-KC with LY294002. Accordingly, LY294002 (10 and 20 μM) attenuated the 7-KC-induced increase of IL-8 expression in endothelial cells as analyzed by RT-PCR (Figure 7D). However, LY294002 cannot prevent the 7-KC-induced activation of ATM/ATR and Chk1/Chk2 (data not shown).

## DISCUSSION

Cardiovascular diseases are the major life-threatening risks to human populations. oxLDLs such as 7-KC and LPC are major etiologic factors that elevate the risk of cardiovascular diseases, due to toxicity of oxLDLs on cardiovascular cells. In this study, 7-KC showed cytotoxicity to endothelial cells, implicating its involvement in the pathogenesis of cardiovascular diseases *via* injury to endothelial cells. On the contrary, 7-KC stimulates the migration and proliferation of vascular smooth muscle cells *via* activation of epidermal growth factor receptor (EGFR), and PI3K/Akt [12]. These results indicate the differential responses of various vascular cells to 7-KC. The cytotoxic effect of 7-KC is due to its induction of G0/G1 and G2/M cell cycle arrest and apoptosis of endothelial cells at concentrations higher than 20 μg/ml. 5,6-Secosterol induces apoptosis of endothelial cells by activating protein kinase C (PKC) and p53, but 7-KC lacks of this effect [13], suggesting the differential effect of various oxysterols to endothelial cells.

7-KC further promotes apoptosis of microvascular endothelial cells *via* caspases-dependent pathways [14]. We also found that 7-KC induce apoptosis of endothelial cells, implicating the involvement of oxysterols in the pathogenesis of atherosclerosis and other degenerative diseases [15]. The induction of cell cycle arrest and cytotoxicity of endothelial cells by 7-KC can be explained by its suppression of cell cycle-related genes and protein expression such as Cdk1 and cyclin B1 of endothelial cells. Cyclin B1 and Cdk1 are crucial molecules for cellular entering to G2/M phase. 7-KC induced G0/G1 cell cycle arrest in human macrophages [16]. On the contrary, 7-KC inhibits proliferation, but not cell cycle progression of adipose tissue-derived stem cells [17]. 7-KC thus leads to endothelial dysfunction and vascular injury *via* damaging of endothelial cells, inducing cell cycle arrest and apoptosis.

Since ATM/Chk2 and ATR/Chk1 are two different oxidative DNA damage response pathways to maintain genome stability [18], we therefore tested and found that 7-KC stimulated ATM/Chk2 and ATR/Chk1 of endothelial cells, suggesting the induction of oxidative DNA damage by 7-KC. Activation of ATM/Chk2 and ATR/Chk1 leads to cell cycle arrest and provides more time for DNA repair [19]. ATM/Chk2 and ATR/Chk stimulate p53 to repress Cdk1 and cyclin B1, thus inducing cell cycle arrest. They also directly inhibit Cdk1 activity by inactivating cdc25, the phosphatase that activates Cdk1 [19]. While 5,6-Secosterol induces apoptosis of endothelial cells by activating PKC and p53, 7-KC lacks of this effect [13]. On the contrary, 7-KC stimulates p53 in PC12 neuronal cells and U937 mononuclear cells [20, 21] and the expression of p53 can be detected in atherosclerotic plaque [21], In this study, the activation of p-53 by 7-KC may explain its repression of Cdk1 and cyclin B1 to induce cell cycle arrest and apoptosis of endothelial cells. This event is associated with oxidative stress induced by 7-KC, because ROS is involved in various diseased and toxic processes by chemicals. The induction of ROS production of endothelial cells by 7-KC can be important in the pathogenesis of vascular diseases by decreasing cell viability and induction various signaling pathways. 7-KC induces apoptosis of mouse aortic endothelial cells and human umbilical vein endothelial cells *via* increase of ROS production, mitochondrial permeabilization and Ca2+ mobilization [22, 23].

Vascular injury and inflammation may cause endothelial dysfunction, bring about atherosclerosis and cardiovascular diseases [24]. Recruitment of inflammatory cells in the intima is an essential step for the development of atherosclerosis, whereas localized production of chemokines such as IL-8, MCP-1, stromal cells-derived factor-1 (SDF-1) etc. are stimulated in endothelial cells and inflammatory cells [25]. Various e-derived cytokines may enhance hematopoiesis, cellular chemotaxis and recruitment, bone resorption, coagulation, and the acute-phase protein synthesis, and thereby generate many diseases, including, graft rejection, asthma, vasculitis, and sepsis [26]. 7-KC may stimulate IL-6 and IL-8 in retina of photodamaged rats [4].7-KC further induces Akt/PKC-NF-kB, p38 and extracellular signal-regulated protein kinase (ERK) signaling pathways of ARPE-19 retina epithelial cells and provokes cytokine production through p38 and ERK [27]. However, little is known about 7-KC on cytokine production of endothelial cells. In this study, 7-KC induces IL-8 expression and secretion of endothelial cells suggesting the contribution of 7-KC in vascular inflammatory response and the diseased processes of cardiovascular disorders.

Limited information is known about PI3K/Akt in 7-KC toxicity. Recent report shows that Akt shows no marked effect on 7-KC-induced cytotoxicity to aortic vascular smooth muscle cells [28] and 7-KC inhibits Akt and glycogen synthase kinase-3beta (GSK3β) phosphorylation in murine oligodendrocytes [29]. 7-KC activates PI3K/mTOR pathway to induce p-glycoprotein expression in Huh7 hepatoma cells [30]. Similarly, 7-KC induces apoptosis of PC12 neuronal cells *via* stimulation of ROS, Akt and caspases [31]. In this study, 7-KC stimulates Akt phosphorylation/activation of endothelial cells. In addition, LY294002 attenuated the 7-KC-induced apoptosis of endothelial cells, suggesting that PI3K/Akt signaling mediates 7-KC cytotoxicity in endothelial cells. Intriguingly, LY294002 attenuated the 7-KC-induced IL-8 expression of endothelial cells, further highlighting PI3K/Akt signaling in 7-KC-induced apoptotic and inflammatory responses in vascular endothelium.

In conclusion, these results reveals the physiological and toxicological effect of 7-KC on the health of cardiovascular system, atherosclerosis and the underlying mechanism. 7-KC shows toxic effects to endothelial cells *via* stimulation of ROS production, cytotoxicity and apoptosis. These events are associated with activation of ATM/Chk2, ATR/Chk1, p53, and PI3K/Akt signaling pathways. These results can be useful for future prevention of atherosclerosis and various cardiovascular diseases related to oxLDLs.

## MATERIALS AND METHODS

### Materials

3-(4,5-dimethylthiazol-2-yl)-2,5-diphenyl tetrazolium bromide (MTT) was purchased from Sigma (Sigma/Aldrich Chemical Company, St. Louis MO, USA). 7-KC was obtained from Cayman (Cayman Chemical Company, Ann Arbor, MI, USA). Cell culture biologicals (Dulbecco's modified Eagle's medium [DMEM], fetal bovine serum [FBS], trypsin/EDTA etc) were obtained from Gibco (Life Technologies, Taipei, Taiwan). Eahy926 (EAHY) endothelial cells were kindly given by Professor Cora-Jean S. Edgell (North Carolina University, NC, USA) [32] and studied in my laboratory before [33, 34]. EAHY cells are hybrid cells from human umbilical vein endothelial cells and have the differentiation function of endothelium and express factor VIII-related antigens [32]. They were cultured in DMEM containing 10% FBS. Enzyme-linked immunosorbant assay (ELISA) kits for IL-8 were obtained from PeproTech (PeproTech Company, Rocky Hill, NJ, USA). Antibodies against Cdk1, cyclin B1, p-ATM, p-ATR, p-Chk1, p-Chk2, p-p53, p-Akt and glyceraldehyde 3-phosphate dehydrogenase (GAPDH) were from Santa Cruz (Santa Cruz Biotechnology Inc., Dallas, Texas, USA).

### Cytotoxicity of 7-KC on Endothelial Cells

Briefly, 5 × 105 EAHY cells were seeded onto 6-well culture plates. After 24 h, culture medium is changed and then various amounts of 7-KC (5-40 μg/ml) or dimethylsulfoxide (DMSO, solvent control) were added. Cells are further incubated for 3 days. Culture medium was collected for ELISA. Then fresh medium containing MTT (final 0.5 mg/ml) was added into each well and cells were cultured for further 2 h. The insoluble formazan generated by viable cells was dissolved in DMSO and read against reagent blank (DMSO) at a wavelength of 540 nm by a microplate reader as before [33, 34] for estimation of cell viability. Results were expressed as Mean ± SEM (% of control).

### Effect of 7-KC on Cell cycle progression

Cell cycle analysis: Briefly 5 × 105 EAHY cells were seeded onto 6-well culture plates. After 24 h, culture medium was changed and then various amounts of 7-KC (final 10, 20, 30, 40, 50 μg/ml) or DMSO were added. Cells were incubated for 3 days. Changes in cell cycle distribution of endothelial cells were investigated by propidium iodide (PI) staining of DNA contents of cells by flow cytometry [35, 36]. Briefly, both floating and attached cells were collected together, re-suspended and fixed for 30 min in 70% ice-cold ethanol containing RNase (2 mg/ml). Cells were then washed with phosphate-buffered saline (PBS) and finally stained with PI (40 μg/ml) for 10 min. The PI-elicited fluorescence of individual cell was measured by a FACSCalibur Flow Cytometer (Becton Dickinson, Worldwide Inc., San-Jose, CA, USA). The wavelength of laser excitation was set at 488 nm and the emission collected was set at greater than 590 nm. The FL2 fluorescence was collected in a linear/log scale fashion. A total of 10,000 cells were analyzed for each sample. The percentage of cells residing in G_0_/G_1_ phase, S phase, G_2_/M and sub-G_0_/G_1_ phase were measured using standard ModiFit software and CELL QUEST programs, respectively.

### Effect of 7-KC on apoptosis of endothelial cells - PI+Annexin V dual fluorescent staining flow cytometry

Briefly 5 × 105 EAHY cells were seeded onto 6-well culture plates. After 24 h, culture medium was changed and then various amounts of 7-KC (final 5, 10, 20, 30, 40 μg/ml) or DMSO were added. Cells were further incubated for 24 hours. Then both the floating and attached cells were harvested. Cells were then washed with PBS, resuspended in 400 μl HEPES (10 mM HEPES-NaOH, pH 7.4, 140 mM NaCl, 2.5 mM CaCl_2_) solution, and the Annexin V-FITC (Becton Dickson)/ PI (50 μg/ml) staining solution was added in the dark for 30 min. The Annexin V-FITC and PI fluorescence of cultured cells were analyzed by FACSCalibur Flow Cytometry immediately as described before [35]. In each analysis, 15,000 events were recorded.

### Reverse transcriptase-Polymerase chain reaction (RT-PCR)

Generally 1.5 × 106 EAHY cells were inoculated onto 10-cm culture dishes and exposed to 7-KC or DMSO for 24h. Total RNA was isolated and subjected for analysis of Cdk1, cyclin B1, IL-8 and beta-actin genes expression by RT-PCR procedures as before [35, 36]. In short 3 μg of denatured RNA was reverse transcribed in a total mixture 45 μl comprising 4 μl of random primer (500 μg/ml), 8 μl of dNTP (2.5 mM), 4.5 μl of 10x RT buffer, 1 μl of RNase inhibitor (40 U/μl), 0.5 μl of RT (21 U/μl) and double distilled water at 42oC for 90 minutes in a thermal cycler. Then same amounts of generated cDNA product were used for further PCR amplification in a reaction mixture comprising 5 μl of 10x Super TAQ buffer, 4 μl of 2.5 mM dNTP, 1 μl of each specific primer, 0.2 μl of Super TAQ enzyme (2 U/μl), and double distilled water. The primers' sequence for beta-actin (BAC): AAGAGAGGCATCCTCACCCT and TACATGGCTGGGGTGTTGAA (218 bp), Cdk1: GGGGATTCAGAAATTGATCA and TGTCAGAAAGCTACATCTTC (288 bp), cyclin B1: AAGAGCTTTAAAC TTTGGTCTGGG and CTTTGTAAGTCC TTGATTTACCATG (317 bp), and IL-8: CACAAGAGCCAGGAAGAAAC and CACAAGAGCCAGGAAGAAAC (459 bp). The amplification procedure for these evaluated genes included 20-35 cycles of PCR, denaturing at 94°C for 1 min, annealing at 55°C for 1 min, and extension at 72°C for 1 min. This was followed by a final incubation at 72°C for 7 min. The PCR products were loaded for 1.8% agarose gel electrophoresis, and finally DNA was stained with ethidium bromide for photograph taking.

### Western blotting

Generally 1.5 × 106 EAHY cells were inoculated onto 10-cm culture dishes and exposed to various concentrations of 7-KC or DMSO for 24h. Cell lysates were prepared by dissolving cells in lysis buffer (10 mM Tris-HCl, pH 7; 140 mM sodium chloride; 3 mM magnesium chloride; 0.5% NP-40; 2 mM phenylmethylsulfonyl fluoride; 1% aprotinin; and 5 mM dithiothreitol) and the same amounts of proteins (20-50 μg/ml) were loaded to 12.5% sodium dodecyl sulfate-polyacrylamide gel electrophoresis (SDS-PAGE) for protein separation and transferred to a polyvinylidene fluoride (PVDF) membrane. The membrane was blotted first with primary antibodies against Cdk1, cyclin B1 and GAPDH for 2 hr as described previously [36, 37]. This was followed by incubation with respective horseradish peroxidase-link secondary antibodies (Jackson ImmunoResearch Laboratories, West Grove, PA, USA) for 1 hr. After rinsed the membrane with buffer, Enhanced chemiluminescence (ECL) reagents (Amersham, Piscataway, NJ, USA) were added and the chemiluminescence was detected by exposure of membranes to Fuji films for 30 sec to 10 min. The intensity of GAPDH bands was used as control.

### Immunofluorescent microscope observation of p-ATM, p-ATR, p-Chk1, p-Chk2, p-p53 and p-Akt Expression of Endothelial cells after exposure to 7-KC

In brief, 1 × 105 EAHY cells were seeded on the sterile coverslips in a 24-well plate in DMEM and 10% FBS. After 24 hours, they were incubated in DMSO or 20 μg/ml of 7-KC for further 24 hours. Medium was then aspirated, and cells were further rinsed with PBS and fixed in 4% paraformaldehyde for 20 min. EAHY cells were rinsed with PBS, permeabilized with 2% Triton X-100, treated by 0.3% v/v H_2_O_2_ for 20 minutes. After further washed by PBS, cells were blocked in 5% bovine serum albumin (BSA) for 1 hr and then incubated in primary antibodies (p-ATM, p-ATR, p-Chk1, p-Chk2, p-p53, p-Akt) (1:1000, v/v) at room temperature for overnight. Following PBS wash, cells were incubated in respective secondary antibody (FITC- or TRITC-conjugated) in the dark for 1 hr and counterstained with 4',6-diamidino-2-phenylindole (DAPI, 1:1000) for 30 min. Finally the cell coverslips were mounted and observed/photographed by an Olympus IX71 inverted microscope and DP Controller/Manager software (Olympus Corporation, Tokyo, Japan) [36].

### Enzyme-linked immunosorbant assay (ELISA) of IL-8 production of EAHY cells

Briefly 5 × 105 EAHY cells were seeded onto 6-well culture plates. After 24 h, culture medium was changed with fresh medium containing various amounts of 7-KC (final concentrations of 5, 10, 20, 30, 40 μg/ml) or DMSO for further 3 days. Culture medium was collected for ELISA analysis of IL-8 following the procedures of assay kits. Cell layer was used for MTT and cell cycle analysis.

### Role of PI3K/Akt signaling pathways in 7-KC-induced alterations of endothelial cells

In some experiments, endothelial cells (5 × 105 cells/well, in 6-well culture) were pretreated by LY294002 (a PI3K/Akt signaling inhibitor) or DMSO for 30 min before addition of 7-KC, and cells were further co-incubated for 3 days. Cellular apoptosis was measured by PI/Annexin V dual fluorescent flow cytometry as described above. IL-8 mRNA expression was studied by RT-PCR as described above.

### Statistical analysis

Three or more independent experiments were conducted. If necessary, paired *t*-test was used for statistical analysis of the difference between groups. A *p* value < 0.05 was considered to have statistically significant difference.
